# Template-Free Electrochemical Deposition of t-Se Nano- and Sub-micro Structures With Controlled Morphology and Dimensions

**DOI:** 10.3389/fchem.2020.00785

**Published:** 2020-08-31

**Authors:** Saba Seyedmahmoudbaraghani, Sooyoun Yu, Jaehong Lim, Nosang V. Myung

**Affiliations:** ^1^Department of Chemical and Environmental Engineering, University of California, Riverside, Riverside, CA, United States; ^2^Department of Materials Science and Engineering, Gachon University, Seongnam-Si, South Korea; ^3^Department of Chemical and Biomolecular Engineering, University of Notre Dame, Notre Dame, IN, United States

**Keywords:** electrodeposition, tetragonal, selenium, nanowire, nanotube

## Abstract

Selenium, depending on its crystal structure, can exhibit various properties and, as a result, be used in a wide range of applications. However, its exploitation has been limited due to the lack of understanding of its complex growth mechanism. In this work, template-free electrodeposition has been utilized for the first time to synthesize hexagonal-selenium (t-Se) microstructures of various morphologies at 80°C. Cyclic voltammetry (CV) and linear sweep voltammetry (LSV) revealed 5 reduction peaks, which were correlated with possible electrochemical or chemical reaction related to the formation of selenium. Potentiostatic electrodeposition using 100 mM SeO_2_ showed selenium nanorods formed at−0.389 V then increased in diameter up to −0.490 V, while more negative potentials (-0.594 V) induced formation of sub-micron wires with average diameter of 708 ± 116 nm. Submicron tubes of average diameter 744 ± 130 nm were deposited at −0.696 V. Finally, a mixture of tubes, wires, and particles was observed at more cathodic potential due to a combination of nucleation, growth, dissolution of structures as well as formation of amorphous selenium via comproportionation reaction. Texture coefficient as a function of applied potential described the preferred orientation of the sub-microstructures changed from (100) direction to more randomly oriented as more cathodic potentials were applied. Lower selenium precursor concentration lead to formation of nanowires only with smaller average diameters (124 ± 42 nm using 1 mM, 153 ± 46 nm using 10 mM SeO_2_ at −0.389 V). Time-dependent electrodeposition using 100 mM selenium precursor at −0.696 V explained selenium was formed first as amorphous, on top of which nucleation continued to form rods and wires, followed by preferential dissolution of the wire core to form tubes.

## Introduction

Chalcogen microstructures are of great research interest due to their broad range of applications, such as optoelectronics (Qin et al., 2017), gas sensors (Tsiulyanu et al., 2001), medicine, energy harvesting, piezoelectrics (Lee et al., 2013), etc. These microstructures have been further exploited to constitute metal chalcogenides, which, combined with finer tuning of electrical properties, can be used in applications such as sensors and solar cells (Yuho et al., 2018).

Selenium (Se), an element of the chalcogen group, exists in three allotropic forms: (1) hexagonal selenium (t-Se), consisting of helical chains of selenium and exhibiting a metallic gray color; (2) monoclinic selenium (m-Se), consisting of Se_8_ rings; and (3) amorphous selenium, consisting of a mixture of disordered chains (Cherin and Unger, 1972; Mehta et al., 2008).

Based on its crystal structures, selenium can exhibit different physical, chemical and electrical properties. For example, hexagonal selenium has high photoconductivity (*e*.*g*., 8 × 10^4^ *S cm*^−1^) (Chen et al., 2009), a low melting point (217°C) (Chen et al., 2009), non-linear optical properties due to its anisotropic crystal structure (Steichen and Dale, 2011), high piezoelectricity ($d_{11}=65\times 10^{-11}\;C/N$)(Mayers et al., 2003), catalytic activity toward organic hydration and oxidation reactions (Xiong et al., 2006), and high reactivity leading to so many functional materials including Ag_2_Se (Tian et al., 2017), CdSe (Jeong et al., 2005; Sobhani and Salavati-Niasari, 2014), ZnSe (Zhang et al., 2016), PbSe (Salavati-Niasari et al., 2013), SnSe (Zhao et al., 2014), and NiSe (Salavati-Niasari and Sobhani, 2013; Hussain and Hussain, 2019). Such properties make Se a promising candidate for applications in photocells, photographic exposure meters, solar cells, semiconducting rectifiers, xerographic copying machines (Zhu and Hu, 2004), gas sensors (Norio and Tsukio, 2011) and Li-Se batteries (Gu et al., 2018). Selenium also has extensive applications in glass decolorization, rubber vulcanizing agent, and lubricant manufacturing. (Shamberger, 1981).

Synthesis methods of selenium structures include photocatalytic, pulsed laser ablation, vapor deposition, hydrothermal, solvothermal, microwave-assisted, and electrochemical. These methods have been utilized in earlier works to achieve various morphologies including nanorods (Cao et al., 2011), nanowires (An et al., 2003), nanotubes (Chen and Gao, 2006), and butterfly-like structures (Zeng et al., 2013). Electrochemical deposition (or electrodeposition) uses an electric current to deposit a material onto a conducting surface by electrochemical reduction; it is a particularly attractive method as it not only is simple and cost-effective, but also allows for fine tuning of size, composition, surface structure and thus chemical and electrical properties via precise control of reaction conditions. Furthermore, electrodeposition can have near-ambient operation conditions, whereas many other methods require extreme operation conditions (i.e., very low or high pressure or temperature), expensive instrumentation, long reaction times, or toxic materials. Despite its many advantages, electrochemical synthesis of selenium microstructures has not been heavily present in recent research works due to the lack of understanding of the complex process of Se deposition. The electroreduction of Se(IV) in aqueous solution, for example, depends on several factors, such as (i) sensitivity of the substrate to the precursor; (ii) underpotential deposition; (iii) semiconductor compound formation; and (iv) coupled chemical reaction. (Wei et al., 1994; Cabral et al., 2010) It is therefore critical to understand the effect of such factors in order to precisely control its structure for specific applications. Recently Saji and Lee (2013) has done an extensive review on the electrochemistry of selenium. However, there are no papers correlating the influence of selenium electrochemistry on the deposition of selenium structures.

In this work, various selenium sub-microstructures were fabricated via template- and surfactant-free electrodeposition, and systematic electroanalytical studies were performed to correlate the formation of the sub-microstructures with the electrochemistry of selenium deposition. The effect of electrolyte composition and applied deposition potential on morphology and crystal structure were investigated.

## Experimental

### Precursor Solution Preparation

All precursor solutions were prepared by dissolving various amounts of SeO_2_ (99.8%, ACROS ORGANICS) in Millipore purified water. The pH of the solutions was adjusted by the addition of H_2_SO_4_ (Certified ACS Plus, Fisher Scientific).

### Linear Sweep Voltammetry

Linear sweep voltammetry (LSV) experiments were performed from +1.5 to −1.4 V vs. Ag/AgCl using a Bio-Logic VMP-3 potentio/galvanostat, while the SeO_2_ concentration was varied from 1 to 100 mM. The scan rate and reaction temperature were fixed at 2 mV/s and 80°C, respectively. LSV at lower bath temperatures of 25 and 60°C were performed as well, but no crystalline selenium formed at 25°C, while the deposits at 60°C were not well crystallized. (Figure A1) Thus, only the results obtained at 80°C were discussed in this work. A conventional three-electrode cell was used in all electrodeposition experiments with titanium (Ti)-gold (Au)-coated (50/200 nm) silicon wafer as the working electrode (area= 0.79 cm^2^), platinum-coated titanium strip as the counter electrode, and a saturated Ag/AgCl (4 M KCl) as the reference electrode (Figure A2). The cell volume was fixed at 100 mL and all the experiments were done in air with no light exposure to prevent photoexcitation of selenium.

### Electrodeposition of Selenium

With the same 3-electrode cell, selenium sub-microstructures were potentiostatically electrodeposited for 2.5 h at a fixed pH of 1.5 and temperature of 80°C, while the SeO_2_ concentration was varied from 1 to 100 mM, and the applied potential from −0.389 to −0.854 V, as determined from the LSV.

Time-dependent experiments were conducted to monitor the growth of selenium structures. Samples were collected at different times (5, 15, 30, 60, and 120 min) of the electrodeposition in the aforementioned three-electrode cell at 80°C with varied selenium precursor concentrations (1, 10, and 100 mM) and applied potentials (from−0.389V to−0.854 vs. Ag/AgCl).

### Characterization of Selenium Sub-microstructures

The morphology and crystal orientation of selenium sub-microstructures were investigated by scanning electron microscopy (SEM, TESCAN VEGA3 and FEI Nova NanoSEM450) and X-ray diffraction (XRD, PANalytical Empyrean) with copper (λ = 1.5405 Å) as anticathode and 0.026-degree increments from 20 to 80°. The average grain size was calculated using the Scherrer equation in (100), (101), and (102) directions. The diameter of the structures was measured from the SEM images utilizing the ImageJ software (30 structures were measured for each SEM image related to every sample).

## Results and Discussion

### Electrochemistry of Selenium in an Acidic Media

The general mechanism of electrodeposition of selenium has been studied and summarized in various works (Andrews and Johnson, 1975; Jarzabek and Kublik, 1980; Kazacos and Miller, 1980; Wei et al., 1994; Lister et al., 1995; Alanyalioglu et al., 2004; Santos and Machado, 2004; Solaliendres et al., 2007; Cabral et al., 2010; Lai et al., 2010). Common possible group of reactions are suggested during the electrode polarization. First, selenium dioxide (SeO_2_) turns into seleneous acid when dissolved in water according to the following reaction:

$$\begin{array}{l} SeO_{2}+H_{2}O\to H_{2}SeO_{3}\;\left(\text{Reaction }1\right) \end{array}$$

The seleneous acid is then reduced by either 4- or 6-electron reduction pathway:

$$\begin{array}{l} H_{2}SeO_{3}+4H^{+}+4e^{-}\to Se+3H_{2}O\;\left(\text{Reaction }2\right) \end{array}$$

$$\begin{array}{l} H_{2}SeO_{3}+6H^{+}+6e^{-}\to H_{2}Se+3H_{2}O\;\left(\text{Reaction }3\right) \end{array}$$

Furthermore, the deposited selenium can be reduced to H_2_Se at more negative potentials shown by the following reaction:

$$\begin{array}{l} Se+2H^{+}+2e^{-}\to H_{2}Se\;\left(\text{Reaction }4\right) \end{array}$$

The presence of H_2_Se in the electrolyte leads to the formation of selenium (amorphous red) through the comproportionation reaction:

$$\begin{array}{l} H_{2}SeO_{3}+2H_{2}Se\to 3Se+3H_{2}O\;\left(\text{Reaction }5\right) \end{array}$$

Although there are a few common proposed electrochemical reduction mechanism reported in prior works, there are different interpretations of the volumetric results.

**Figure 1:**
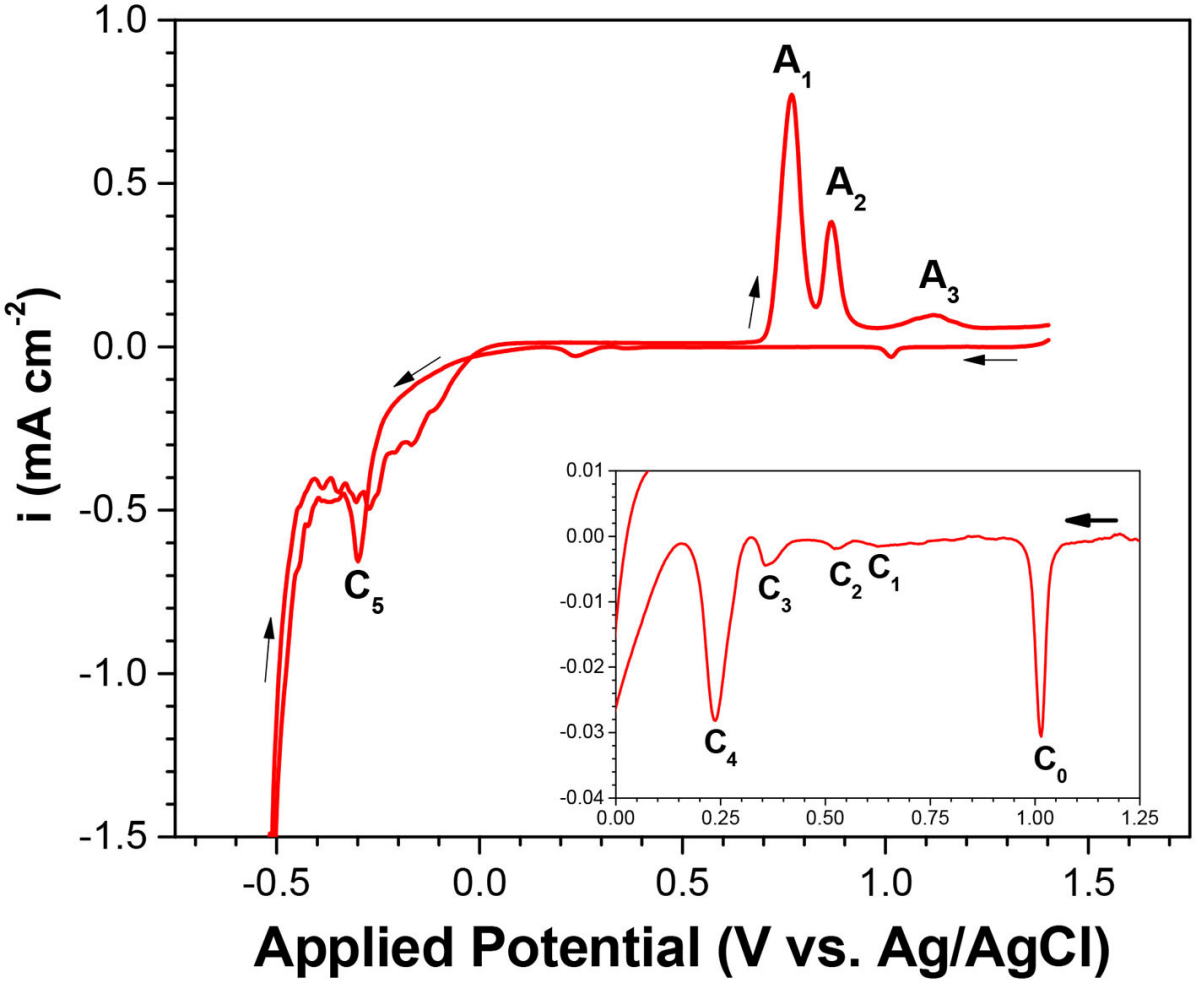
Cyclic voltammogram (CV) of Se electrodeposition at pH of 1.5 and scan rate of 2 mV/s. Temperature was fixed at 80°C with no illumination. Inset shows the expanded y-scale of applied potential from 1.25 to 0 V.

For a comprehensive understanding of the electrochemistry of selenium, cyclic voltammetry of electrolyte containing 1 mM of SeO_2_ was carried out. The potential was swept from 1.4 to −0.8 V in the negative direction and then back to 1.4 V in the positive sweep direction. In the voltammogram, 5 reduction peaks (C_1_-C_5_) and 3 oxidation peaks (A_1_-A_3_) were observed. (Figure 1).

Based on other work and the Pourbaix diagram, available species at pH 1.5 across the potential range were identified and then correlated to each peak. C_1_ to C_3_ exhibited reduction peaks similar to that of underpotential deposition (UPD) of Se(0) onto the gold substrate; C_4_ was related to the bulk deposition of Se(0); and C_5_ corresponded to the formation of H_2_Se, either by reduction of Se(0) to Se(-II) or direct 6-electron reduction of Se(IV) present in the electrolyte. As potential more negative than −0.5 V was applied, the current density increased drastically due to the hydrogen evolution reaction as the dominant reaction:

$$\begin{array}{l} 2H^{+}+2e^{-}\to H_{2}\left(g\right)\;\left(\text{Reaction }6\right) \end{array}$$

In the reverse scan, the three oxidation peaks were correlated with selenium oxidation, as supported by literature (Kowalik, 2015). The first oxidation peak, A_1_, was correlated with the dissolution of bulk selenium; the second oxidation peak (A_2_) with the dissolution of underpotentially deposited selenium; and the third oxidation peak (A_3_) with the oxidation of selenium, which diffused into the gold electrode. It should be noted that small shifting and overlapping of the peaks were observed compared to other works, which was expected due to different substrate material, electrolyte composition, and some operating (e.g., scan rate) conditions.

To investigate the effect of precursor concentration on each of the cathodic peaks, linear sweep voltammetry was conducted at higher SeO_2_ concentrations (10 and 100 mM), as shown in Figure A3. As the SeO_2_ concentration was increased, the peaks C_1_ to C_3_ shifted to more positive potentials. At the highest concentration of SeO_2_ (100 mM), peak C_3_ combined with peak C_2_, while peak C_1_ appeared as a shoulder to peak C_2_. This was probably because more precursor was available at higher concentrations, reducing the amount of energy required to drive the reactions, and as a result, causing all three peaks to shift to more positive potentials. Meanwhile, the change in peak potential of C_1_ was not as drastic as that of C_2_ or C_3_, as these peaks were representative of the underpotential deposition of Se.

Furthermore, concentration-dependent LSV was used to determine the number of electrons transferred and confirm the assignment of the peaks. Assuming the surface concentration of the adsorbed selenium did not change over the range of solution activities used in this work, the peak potential of the reduction wave was described by the Nernst equation:

(1) $$\begin{array}{r} E_{p,C1}=E^{0}+2.303\frac{RT}{nF}\log a_{SeO_{2}} \end{array}$$

**Figure 2:**
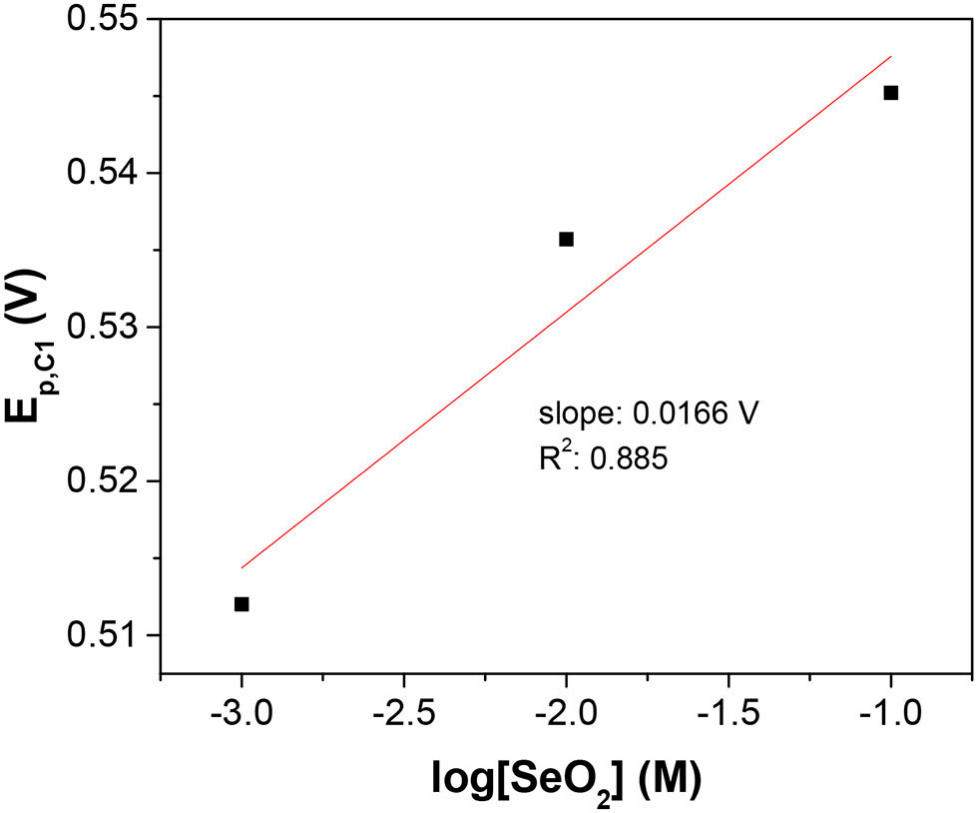
Semi-log plot of C1 peak potential as a function of SeO_2_ concentration. The x-axis is the logarithm of SeO_2_ concentration in the electrolyte. The temperature and pH were fixed at 80°C and 1.5, respectively.

**Figure 3:**
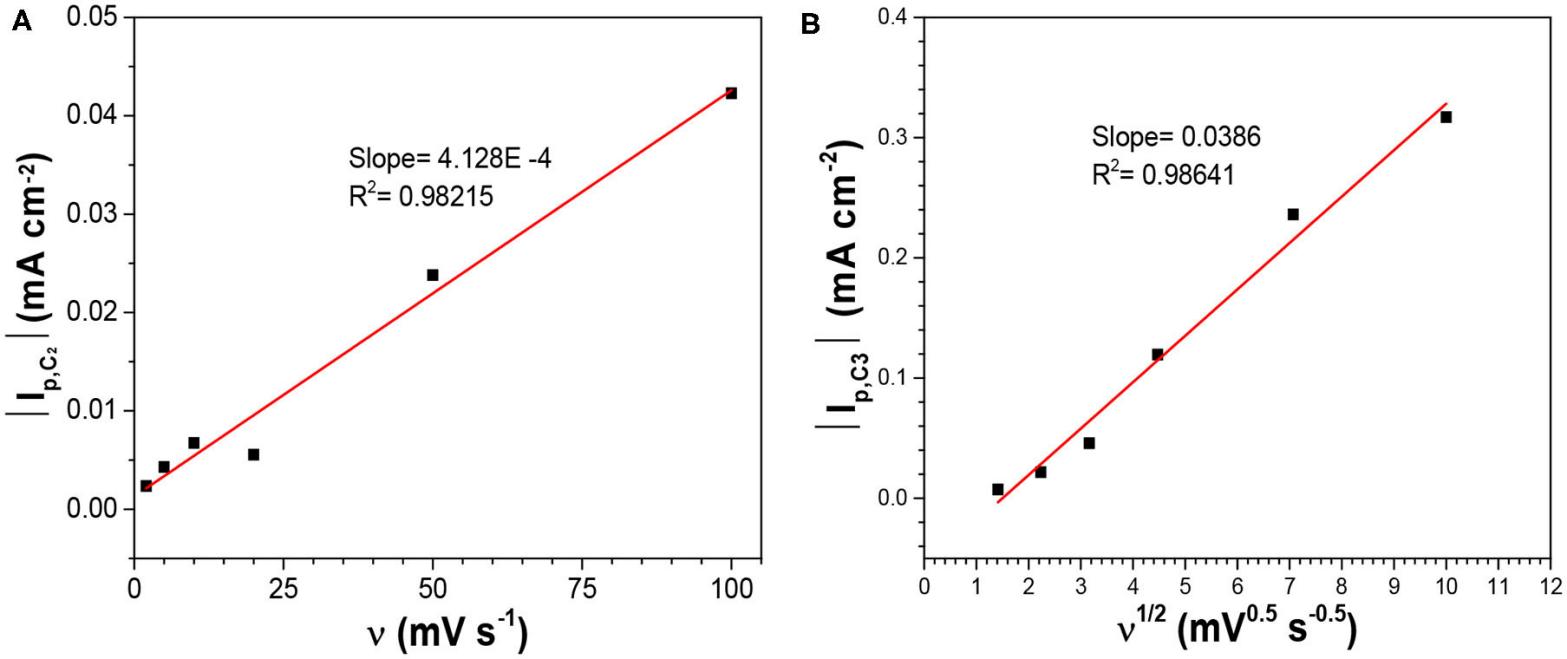
Dependency of the peak currents of **(A)** C_2_, and **(B)** C_3_ on the scan rate in the electrolytes containing 10 mM SeO_2_ at pH1.5 and 80°C without illumination.

Where *n* is the number of electrons transferred. As it was safe to assume that the activity coefficient for the dissolved SeO_2_ would stay constant with the electrolyte compositions in this work, the plot of E_p, C1_ vs. log[SeO_2_] would exhibit a linear relationship with the slope of (0.0701/*n*) at 80°C (Figure 2). The calculated slope was 0.0166 V, which corresponded to the transference of 4 electrons, indicating that peak C_1_ was associated with reaction 2. To better understand the nature of these peaks, scan rate-dependent cyclic voltammetry was done at 10 mM SeO_2_ (Figure A4). Qualitatively, peak potential shift of approximately 50 mV was observed for both peak C_3_ and C_4_ as shown in the inset of Figure A4, indicative of a quasi-reversible or irreversible process, which was also obvious from the very large separation from the three anodic peaks representing the dissolution of deposited Se. Same observation was made for peaks C_1_ and C_2_ (not marked on the inset of Figure A4). The increase in the peak current as a function of scan rates was also confirmed, which were attributed to the higher diffusion flux with higher precursor concentration gradient with higher scan rates, resulting in higher peak currents. The dependency of peak current density on the scan rate was investigated to understand the nature of the peaks C_1_-C_3_, shown in Figure 3. The C_2_ peak current density displayed a linear dependence on the scan rate, indicative of an adsorbed species (2002). This finding suggests the presence of an adsorbed layer of selenite which would then reduce to selenium similar to previous observations (Alanyalioglu et al., 2004). On the other hand, the current density of peak C_3_ was linearly dependent on the square-root of the scan rate, which suggested a diffusion-limited process, according to the Randles-Sevcik equation (2002). However, no relationship with scan rate could be observed for peak C_1_ due to the very small peak current densities associated with this peak but since the peak potential is more positive than C_2_ and C_3_, it was safe to assume that this peak was indicative of UPD. All three peaks were therefore associated with the UPD of selenium. The presence of 3 peaks for the UPD of selenium might be an indication of additional coverage to form a sub-monolayer of selenium.

The coverage of selenium sub-monolayer was calculated based on the number of electrons transferred as well as the charge associated with the UPD of selenium, which was calculated using the area underneath each of peaks C_1_-C_3_. The calculations did not account for the surface roughness. As all three peaks attributed to the UPD of Se, coverage of Se was calculated for each peak and then added together to determine the total coverage of Se as a result of UPD. As seen in Figure A5, coverage of selenium sub-monolayer increased as a function of SeO_2_ concentration. At 1 mM SeO_2_, only 0.17 monolayer was deposited, while 0.2 and 0.43 monolayer were deposited at 10 and 100 mM, respectively, which was consistent with literature data (Alanyalioglu et al., 2004).

In the CV presented in Figure 1, peak C_4_ represented the bulk deposition of Se (0) due to the reduction of Se(IV) (Reaction 2). At 10 mM (Figure A3A), peak C_4_ divided into a peak (C_4−1_) and a broad shoulder at more negative potentials. This peak appears as 3 distinct peaks at the highest concentration of SeO_2_, 100 mM (Figure A3B). This could be attributed to the slow and sluggish kinetics of selenium electrodeposition. It was also shown that peak C_4_ shifted to more cathodic potentials at higher SeO_2_ concentrations, since the bulk deposition of selenium was a mass transport limited reaction.

Peak C_5_ was correlated the formation of H_2_Se due to either the reduction of Se(0) to Se(-II) (Reaction 3) or the direct reduction of Se(IV) present in the solution (Reaction 4). It was evident that the peak position shifted to more negative potentials with the increase in SeO_2_ concentration. This was due to the comproportionation reaction (Reaction 5), which led to the formation of amorphous (red) Se(0) that could passivate the electrode surface. At lower concentrations, there was only a single peak concerning the 2-electron reduction of Se(0) and direct 6-electron reduction of Se(IV). At higher concentrations (100 mM SeO_2_), two reduction peaks were observed related to each of these reactions. Since 6-electron reduction happens at more cathodic potentials than the 2-electron reduction of Se (0) (E^0^ = +0.316 and−0.445 V vs. Ag/AgCl, respectively), peak C_5−1_ was associated with the 6-electron reduction. However, further investigation is required to confirm this.

### Characterization of Selenium Electrodeposits

With an insight of selenium electrochemistry from the CV and LSV, potentiostatic experiments were carried out to elucidate the effect of experimental conditions on the crystallinity and morphology of the electrodeposited selenium.

#### Effect of Applied Potential

**Figure 4:**
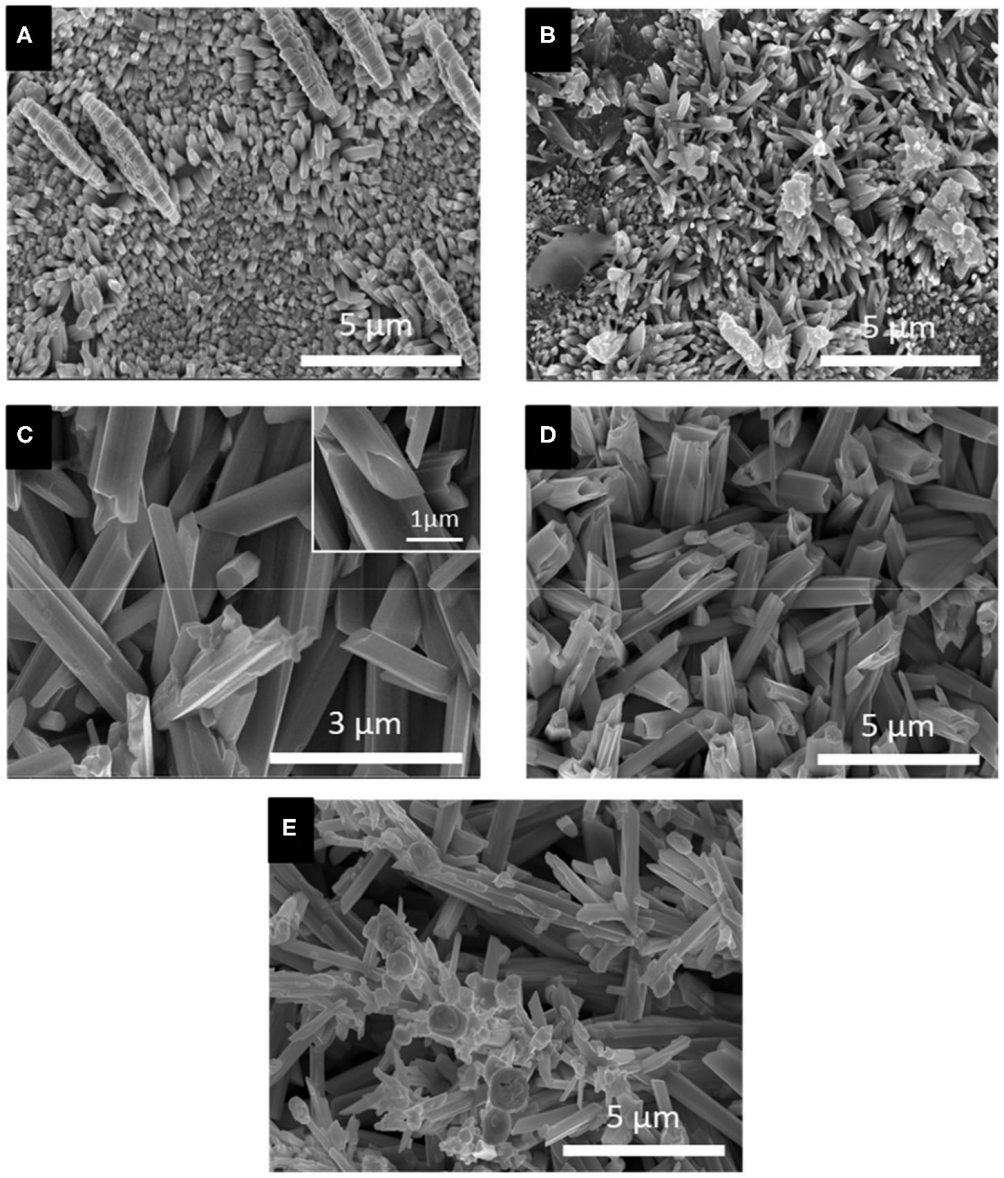
SEM images of electrodeposited selenium from 100 mM SeO_2_ at **(A)** −0.389 V, **(B)** −0.490 V, **(C)** −0.594 V, **(D)** −0.696 V, and **(E)** −0.854 V. Deposition time and temperature were fixed at 150 min and 80°C, respectively.

Based on the LSV and CV studies, 5 different applied potentials were chosen to monitor the formation of Se structures more closely. At more positive potentials where the bulk deposition of selenium occurred according to the CV and LSV curves, no deposits were observed due to slow kinetics of the deposition even after 150 min. For this reason, the deposition potentials chosen were in more negative range before (−0.389 V), during (−0.490 V), and after (−0.594 V) the formation of H_2_Se. Two more negative potentials were chosen to monitor the change in morphology of Se deposits shortly before (−0.696 V) and after (−0.854 V) hydrogen gas evolution. The morphology of the Se deposits from 100 mM SeO_2_ were studied via SEM, as shown in Figure 4. The average diameter of the Se deposits at each potential was calculated over 30 unique measurements using Image-J software. It is worth noting that the morphology was uniform on the surface of the samples.

Figure 4A, corresponding to selenium deposited at −0.389 V vs. Ag/AgCl, showed selenium nanorods with the average diameter of 200 ± 40 nm, along with some selenium microplates. According to the linear sweep voltammogram, the deposition at this potential would only be due to the bulk reduction of Se(IV) to Se(0) (Reaction 2). When more negative potential of −0.490 V vs. Ag/AgCl was applied (Figure 4B), longer nanorods were deposited, attributed to the higher rate of bulk deposition. The average diameter of the nanorods increased slightly to 213 ± 41 nm. In addition, the comproportionation reaction (Reaction 5) along with the bulk deposition caused the secondary nucleation on the Se nanorods, leading to the formation of flower-like structures.

At −0.594 V vs. Ag/AgCl (Figure 4C), the bulk deposition rate increased even further, leading to the formation of selenium sub-micron wires, with a drastically increased average diameter of 708 ± 116 nm. The deviation in the average diameter of the wires could be explained by the combination of continued nucleation from the bulk deposition of selenium—resulting in smaller sized structures—and growth of previously deposited selenium structures. Additionally, the wires formed at this potential exhibited a hexagonal cross-sectional area. This could be described by the fact that, at 80°C, t-Se structures were anticipated to deposit, which consist of helical chains that are formed by the period of three Se atoms (Yan et al., 2009). Furthermore, as highlighted in the inset of Figure 4C, dents on the tip of the nanowires were observed, which could lead to the formation of nanotubes by hollowing out of the nanowires. This might be attributed to selenium cathodic dissolution (i.e., formation of H_2_Se), the rate of which was significant at this potential. The hollow part of these nanotubes, however, was not observed all the way through to the base of the wires. By applying more negative potentials (i.e., −0.696 V vs. Ag/AgCl), the dissolution reaction was even more dominant, leading to the formation of selenium sub-micron tubes with the average diameter of 744 ± 130 nm, as shown in Figure 4D. Amorphous (red) selenium was also formed due to the chemical reaction presented in Reaction 5. At −0.854 V, the electrodeposited structures were a combination of wires, tubes, and particles, with no apparent increase in diameter compared to those deposited at −0.696 V. (Figure 4E) This was because, in addition to rapid deposition of Se(0), the dissolution of Se(0) and deposition of amorphous Se occurred at almost the same rate, preventing the formation of structures with well-defined shape. These results are summarized in Table 1.

**Table 1:** Summary of electrodeposited selenium structures from an electrolyte containing 100 mM SeO_2_.

**Applied Potential (V vs. Ag/AgCl)**	**Structure Diameter (nm)**	**Morphology**
−0.389	200 ± 40	Nanorods and microplates
−0.490	213 ± 41	Longer nanorods and flower-like structures
−0.594	708 ± 116	Sub-microwires
−0.696	744 ± 130	Sub-microtubes
−0.854	744 ± 130	Combination of wires, tubes, and particles

*Deposition time and temperature were fixed at 150 min and 80°C, respectively*.

**Figure 5:**
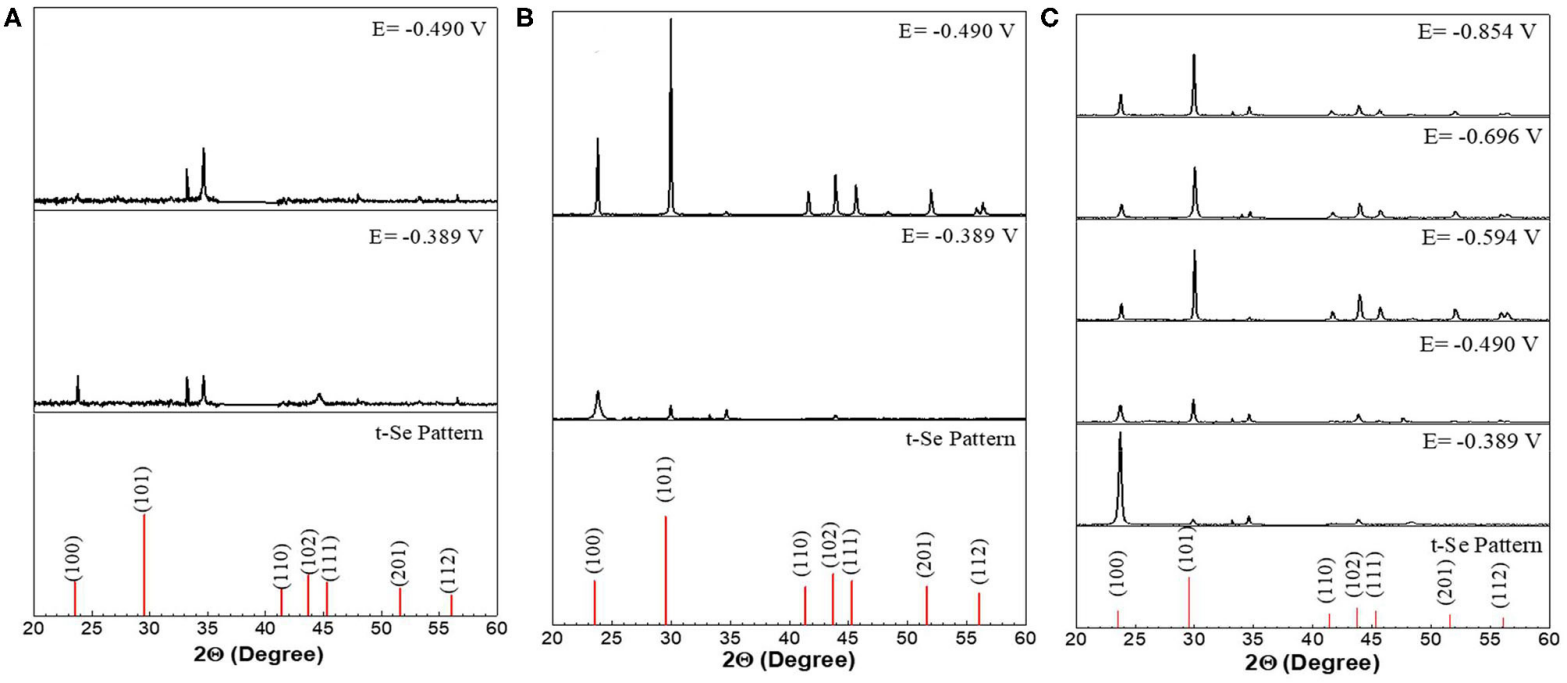
XRD analysis of selenium nanostructures after electrochemical deposition for 150 min at different applied potentials from electrolyte containing different concentration **(A)** 1mM, **(B)** 10mM, **(C)** 100mM of SeO_2_. Electrochemical deposition was done at 80°C. Electrolyte pH and sweep rate were 1.5 and 2 mV/s respectively.

XRD analysis results further confirmed the presence of t-Se structures in the samples collected at all applied potentials and SeO_2_ concentrations (Figure 5). Furthermore, to determine the preferred orientation of the electrodeposited selenium structures, texture coefficient was calculated according to the following equation:

(2) $$\begin{array}{r} T_{hkl}=\frac{I_{hkl}/I_{hkl}^{0}}{\left(1/n\right)\sum \left(I_{hkl}/I_{hkl}^{0}\right)} \end{array}$$

**Figure 6:**
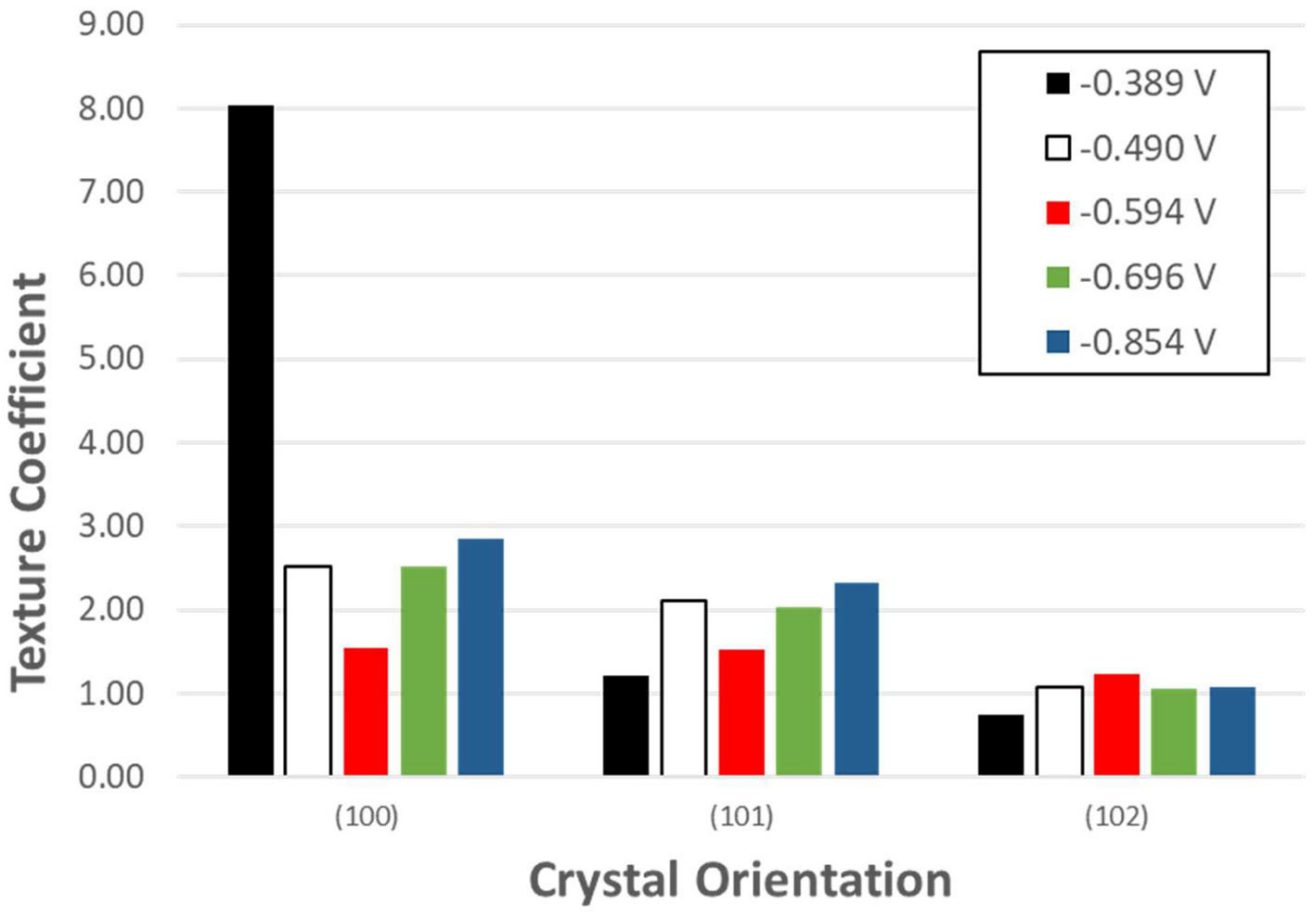
Texture coefficient of potentiostatically electrodeposited selenium nanostructures from 100 mM SeO_2._ The deposition time was fixed at 150 min.

Where T_hkl_ is the texture coefficient, and I_hkl_ and I${}_{\text{hkl}}^{0}$ are the intensity of the diffraction peak in the pattern obtained and the standard, respectively. The calculated texture coefficient for each crystal orientation of Se samples electrodeposited at the five applied potentials are shown in Figure 6.

Se nanorods deposited at −0.389 V vs. Ag/AgCl was highly crystalline, and the preferred orientation of the nanorods was (100). The SEM image of the deposits at this potential also shows nanorods grown in the c-axis direction which is in line with the XRD results. Application of more negative potential (i.e., −0.490 V vs. Ag/AgCl) caused the electrodeposited t-Se sub-microstructures to be more randomly oriented. This might be as a result of formation of new nuclei, which caused the formation of additional structures. SEM images confirm this as well.

In addition, the grain size of the structures was calculated from the XRD results using the Scherrer's equation. It is worth noting that the calculated grain size was drastically smaller than the size of the structures, pointing to their polycrystallinity.

#### Effect of SeO_2_ Concentration

**Figure 7:**
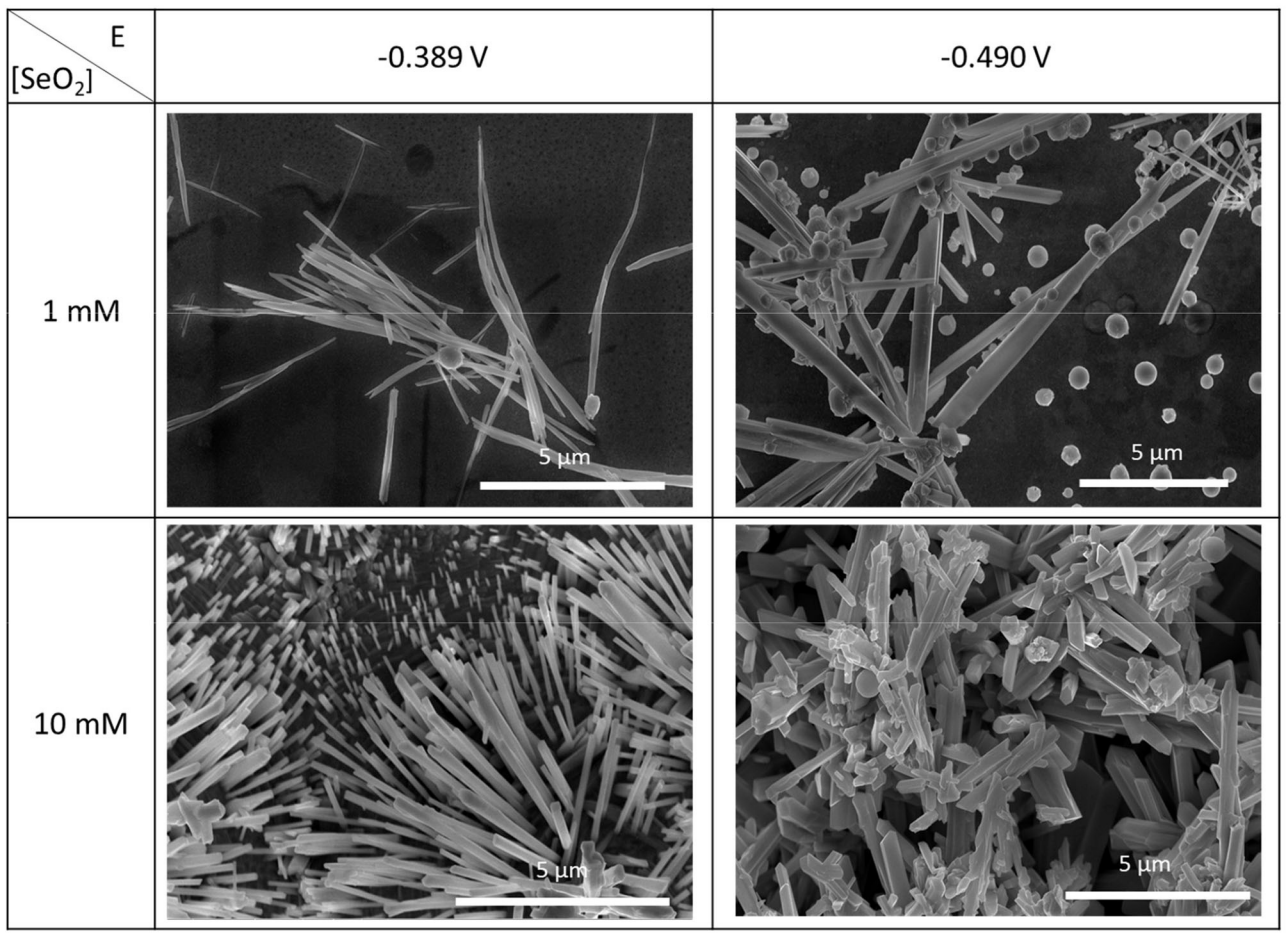
SEM images of electrodeposited selenium from 1 and 10 mM SeO_2_ at −0.389 and −0.490 V. The deposition time and temperature were fixed at 150 min and 80°C, respectively.

To investigate the effect of SeO_2_ concentration on the morphology of the electrochemically deposited selenium structures, same potentiostatic experiments were also conducted in electrolytes containing 1 and 10 mM SeO_2_. Only the two most cathodic potentials were chosen for these lower SeO_2_ concentrations as no further deposits other than the amorphous Se was expected at potentials more negative than −0.490 V, based on the LSV shown in Figure A3. SEM images of the as-deposited selenium structures at these conditions were used to measure the average diameter (Figure 7).

In an electrolyte containing 1 mM SeO_2_, selenium nanowires with the average diameter of 124 ± 42 nm were obtained, while some a-Se particles were also observed due to the comproportionation reaction. When the applied potential was reduced to −0.490 V, the nanowires turned into sub-micro wires and the average diameter increased to 437 ± 113 nm as did the amount of a-Se. By increasing the concentration of selenium precursor by tenfold, selenium nano and sub-microwires also formed at both applied potentials, but at −0.490 V, the surfaces of the wires were not smooth. Furthermore, formation of amorphous selenium particles was again due to the chemical reaction between H_2_Se and H_2_SeO_3_ that were already present in the electrolyte. The diameter of selenium wires deposited at −0.389 and −0.490 V were 153 ± 46 and 584 ± 149 nm, respectively. It was apparent that the diameter of the selenium sub-microwires increased as a function of the SeO_2_ concentration at fixed potentials (Table 2). It is important to note that at −0.490 V, significantly larger structures have deposited using 10 mM SeO_2_ compared to 100 mM. This was attributed to the fact that H_2_Se formation occurred at this potential in 10 mM SeO_2_–but not in 100 mM SeO_2_–at which the bulk deposition of Se continued, leading to larger Se structures. These results were supported by the XRD analysis (Figure 5). The calculated texture coefficient was also in line with what was observed at 100 mM SeO_2_.

**Table 2:** Summary of electrodeposited selenium structures from an electrolyte containing 1 and 10 mM SeO_2_.

**[SeO_**2**_] (mM)**	**Applied Potential (V vs. Ag/AgCl)**	**Structure diameter (nm)**
1	−0.389	124 ± 42
	−0.490	437 ± 113
10	−0.389	153 ± 46
	−0.490	584 ± 149

*Deposition time and temperature were fixed at 150 min and 80°C, respectively*.

### Growth Process of t-Se Sub-microstructures

**Figure 8:**
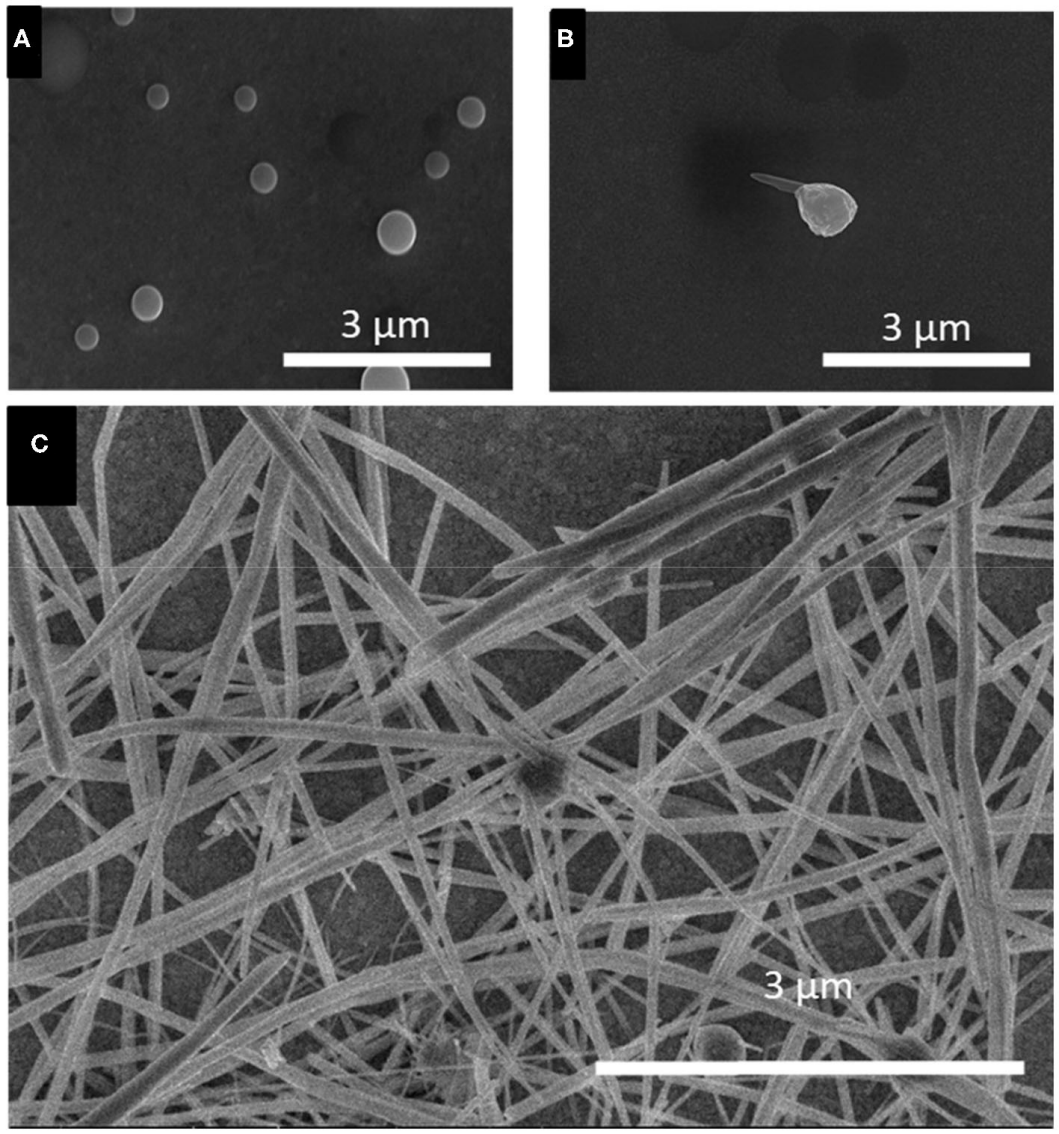
SEM images of selenium electrodeposited for **(A)** 15 min, **(B)** 30 min, and **(C)** 60 min. Applied potential and SeO_2_ precursor concentration were fixed at −0.389 V and 1 mM, respectively. The deposition temperature was fixed at 80°C.

The growth process of the t-Se sub-microstructures was elucidated via morphological change over the duration of electrodeposition at −0.389 V using 1 mM SeO_2_, observed by SEM images of the Se deposits collected at different times (Figure 8). After 15 min (Figure 8A), spherical a-Se particles (average diameter: 1.85 ± 0.3 μm) were formed. After 30 min (Figure 8B), small t-Se nanocrystallites were observed on the surface of the a-Se particles. These crystallites served as seeds for the t-Se nanostructures, which grew at the expense of the a-Se particles. At 60 min (Figure 8C), the crystal seed grew further into sub-microstructures (sub-microwires at this condition). When the electrodeposition duration was increased to 120 min, there was no apparent broadening of the diameter of the sub-microwires.

**Figure 9:**
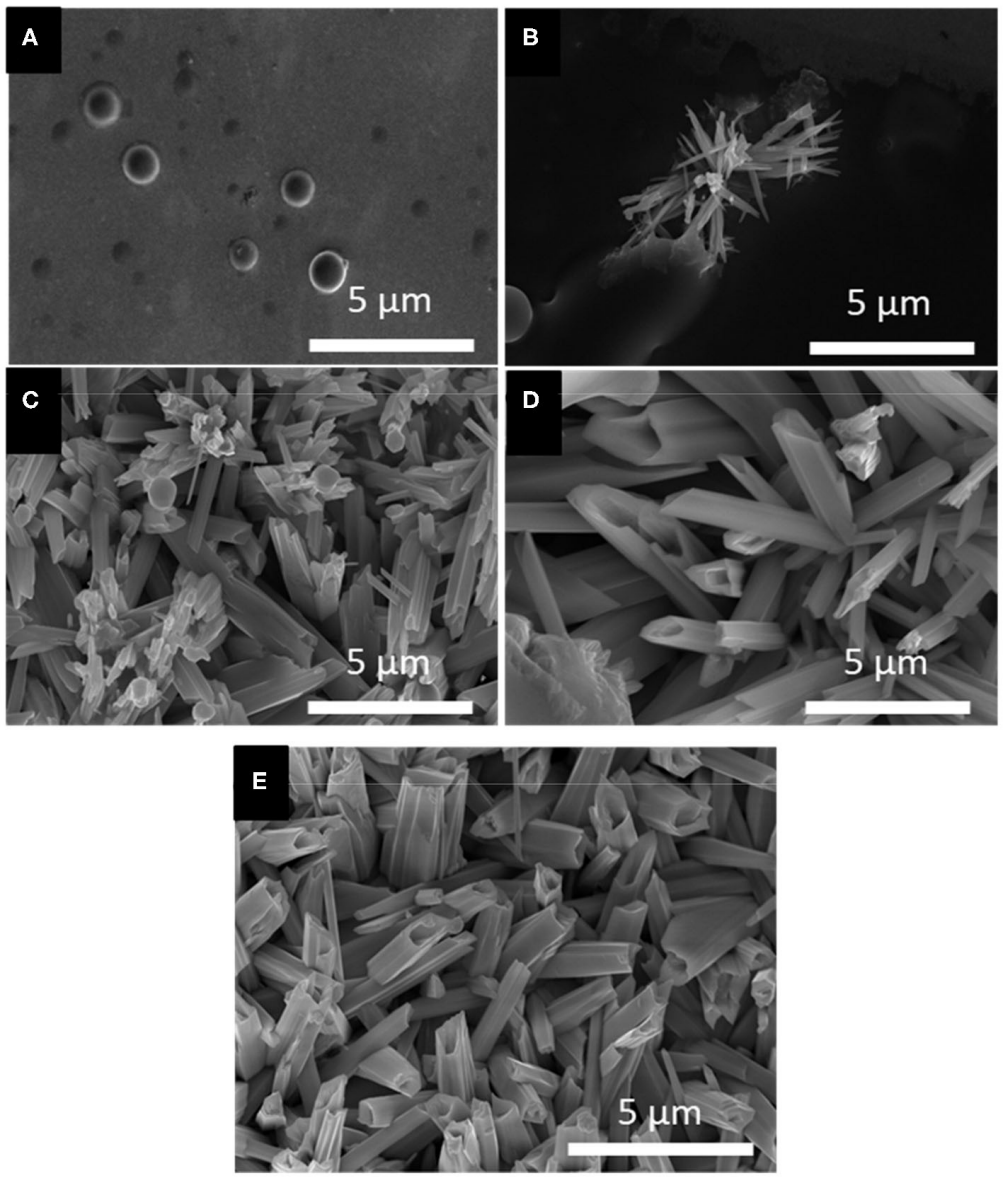
SEM images of selenium electrodeposited for **(A)** 15 min, **(B)** 30 min, and **(C)** 60 min, **(D)** 120 min and **(E)** 150 min. Applied potential and SeO_2_ precursor concentration were fixed at −0.696 V and 100 mM, respectively.

To investigate the growth process of t-Se sub-micron tubes, similar time-dependent experiments were conducted in the solution containing 100 mM SeO_2_, at −0.696 V vs. Ag/AgCl (Figure 9). It was shown that the initial stages of the growth process were similar to that using 1 mM SeO_2_, up to the formation of t-Se sub-microwires. (Figures 9A,B) But after 30 min of deposition, dissolution of Se(0) led to the formation of dents on the tip of the nanowires, eventually creating a void to form t-Se tubes (Figures 9C,D).

## Conclusion

In summary, the complex electrochemistry of selenium was systematically investigated using a series of electroanalytical studies and was determined to be dependent on the precursor concentration in the electrolyte. For the first time, t-Se structures of various morphologies was achieved by electrodeposition without the aid of any template. Potentiostatic electrodeposition at various selenium precursor concentrations revealed that the applied potential as well as the [SeO_2_] changed the governing electrochemical reaction, thereby enabling the fine tuning of morphology and dimensions of the structures. Potentiostatic electrodeposition at 100 mM SeO_2_, specifically, allowed for the precise control of selenium sub-microstructures, from nanorods at−0.389 V to sub-microwires at −0.594 V, and finally sub-micron tubes at −0.696 V and 3-D structures at −0.854 V, with diameters ranging from 200 ± 40 nm to 744 ± 130 nm. Similar electrodeposition at lower selenium precursor concentration (i.e., 10 and 1 mM) exhibited the formation of nanowires only. The diameters of nanowires formed at −0.389 V with 10 and 1 mM SeO_2_ were 153 ± 46 nm and 124 ± 42 nm, respectively. X-ray diffraction analysis confirmed the growth of t-Se crystallites in the c-axis direction, while variation of the applied potential could change the preferred orientation of the structure growth. Finally, the growth mechanism of selenium sub-micro/nanostructures was optically confirmed with time-dependent electrodeposition.

## Data Availability Statement

All datasets presented in this study are included in the article/Supplementary Material.

## Author Contributions

SS conducted most of experiment and draft the manuscript. SY and JL provides their expertise to guide experiment, provided inputs during editing and anlayze the data. NM came up with the idea, obtain the funding and supervise SS and SY. All authors contributed to the article and approved the submitted version.

## Conflict of Interest

The authors declare that the research was conducted in the absence of any commercial or financial relationships that could be construed as a potential conflict of interest. The handling editor declared a past co-authorship with one of the authors JL.

## References

[B1] Alanyalioglu M. Demir U. Shannon C. (2004). Electrochemical formation of Se atomic layers on Au(111) surfaces: the role of adsorbed selenate and selenite. J. Electroanal. Chem. 561, 21–27. 10.1016/j.jelechem.2003.07.016

[B2] An C. Tang K. Liu X. Qian Y. (2003). Large-scale synthesis of high quality trigonal selenium nanowires. Eur. J. Inorg. Chem. 2003, 3250–3255. 10.1002/ejic.200300142

[B3] Andrews R. W. Johnson D. C. (1975). Voltammetric deposition and stripping of selenium(IV) at a rotating gold-disk electrode in 0.1M perchloric acid. Anal. Chem. 47, 294–299. 10.1021/ac60352a005

[B4] Cabral M. F. Pedrosa V. A. Machado S. A. S. (2010). Deposition of selenium thin layers on gold surfaces from sulphuric acid media: Studies using electrochemical quartz crystal microbalance, cyclic voltammetry and AFM. Electrochim. Acta 55, 1184–1192. 10.1016/j.electacta.2009.10.008

[B5] Cao G. S. Juan Zhang X. Su L. Yang Ruan Y. (2011). Hydrothermal synthesis of selenium and tellurium nanorods. J. Exp. Nanosci. 6, 121–126. 10.1080/17458081003774677

[B6] Chen M. Gao L. (2006). Selenium nanotube synthesized via a facile template-free hydrothermal method. Chem. Phys. Lett. 417, 132–136. 10.1016/j.cplett.2005.09.083

[B7] Chen Z. Shen Y. Xie A. Zhu J. Wu Z. Huang F. (2009). l-Cysteine-assisted controlled synthesis of selenium nanospheres and nanorods. Cryst. Growth Des 9, 1327–1333. 10.1021/cg800398b

[B8] Cherin P. Unger P. (1972). Refinement of the crystal structure of [alpha]-monoclinic Se. Acta Crystallogr. Section B 28, 313–317. 10.1107/S0567740872002249

[B9] Gu X. Xin L. Li Y. Dong F. Fu M. Hou Y. (2018). Highly reversible Li–Se batteries with ultra-lightweight N,S-codoped graphene blocking layer. Nano-Micro Letters 10:59. 10.1007/s40820-018-0213-5 30393707 PMC6199105

[B10] Hussain R. A. Hussain I. (2019). Fabrication and applications of nickel selenide. J. Solid State Chem. 277, 316–328. 10.1016/j.jssc.2019.06.015

[B11] Jarzabek G. Kublik Z. (1980). Cyclic and stripping voltammetry of Se(+4) and Se(-2) at carbon electrodes in acid solutions. J. Electroanal. Chem. Interf. Electrochem. 114, 165–177. 10.1016/S0022-0728(80)80445-1

[B12] Jeong U. Kim J.-U. Xia Y. Li Z.-Y. (2005). Monodispersed spherical colloids of Se@CdSe: synthesis and use as building blocks in fabricating photonic crystals. Nano Lett. 5, 937–942. 10.1021/nl050482i 15884898

[B13] Kazacos M. S. Miller B. (1980). Studies in selenious acid reduction and CdSe film deposition. J. Electrochem. Soc. 127, 869–873. 10.1149/1.2129772

[B14] Kowalik R. (2015). The voltammetric analysis of selenium electrodeposition from H_2_SeO_3_ solution on gold electrode. Arch. Metal. Mater. 60:57 10.1515/amm-2015-0009

[B15] Lai Y. Liu F. Li J. Zhang Z. Liu Y. (2010). Nucleation and growth of selenium electrodeposition onto tin oxide electrode. J. Electroanal. Chem. 639, 187–192. 10.1016/j.jelechem.2009.11.026

[B16] Lee T. I. Lee S. Lee E. Sohn S. Lee Y. Lee S.. (2013). High-power density piezoelectric energy harvesting using radially strained ultrathin trigonal tellurium nanowire assembly. Adv. Mater. 25, 2920–2925. 10.1002/adma.201300657 23616287

[B17] Lister T. E. Huang B. M. II. R. D. H. Stickney J.L. (1995). Electrochemical formation of Se atomic layers on Au(100). J. Vacuum Sci. Technol. B 13, 1268–1273. 10.1116/1.587836

[B18] Mayers B. T. Liu K. Sunderland D. Xia Y. (2003). Sonochemical synthesis of trigonal selenium nanowires. Chem. Mater. 15, 3852–3858. 10.1021/cm034193b

[B19] Mehta S. K. Savita C. Sanjay K. Bhasin K. K. Kanjiro T. Hideki S.. (2008). Surfactant assisted synthesis and spectroscopic characterization of selenium nanoparticles in ambient conditions. Nanotechnology 19:295601. 10.1088/0957-4484/19/29/295601 21730604

[B20] Norio A. Tsukio O. (2011). Gas detection of volatile organic compounds using trigonal selenium nanowires. Jpn. J. Appl. Phys 50:015002 10.1143/JJAP.50.015002

[B21] Qin J. Qiu G. Jian J. Zhou H. Yang L. Charnas A.. (2017). Controlled growth of a large-size 2D selenium nanosheet and its electronic and optoelectronic applications. ACS Nano 11, 10222–10229. 10.1021/acsnano.7b04786 28949510

[B22] Saji V. S. Lee C.-W. (2013). Selenium electrochemistry. RSC Adv. 3, 10058–10077. 10.1039/C3RA40678D

[B23] Salavati-Niasari M. Shoshtari-Yeganeh B. Mohandes F. (2013). Schiff-base assisted synthesis of lead selenide nanostructures. Mater. Res. Bull. 48, 1745–1752. 10.1016/j.materresbull.2012.12.076

[B24] Salavati-Niasari M. Sobhani A. (2013). Effect of nickel salt precursors on morphology, size, optical property and type of products (NiSe or Se) in hydrothermal method. Opt. Mater. 35, 904–909. 10.1016/j.optmat.2012.11.004

[B25] Santos M. C. Machado S. A. S. (2004). Microgravimetric, rotating ring-disc and voltammetric studies of the underpotential deposition of selenium on polycrystalline platinum electrodes. J. Electroanal. Chem. 567, 203–210. 10.1016/j.jelechem.2003.12.026

[B26] Shamberger R. J. (1981). Selenium in the environment. Sci. Total Environ. 17, 59–74. 10.1016/0048-9697(81)90108-X 7010600

[B27] Sobhani A. Salavati-Niasari M. (2014). Synthesis and characterization of CdSe nanostructures by using a new selenium source: Effect of hydrothermal preparation conditions. Mater. Res. Bull. 53, 7–14. 10.1016/j.materresbull.2014.01.028

[B28] Solaliendres M. O. Manzoli A. Salazar-Banda G. R. Eguiluz K. I. B. Tanimoto S. T. Machado S. A. S. (2007). The processes involved in the Se electrodeposition and dissolution on Au electrode: the H2Se formation. J. Solid State Electrochem. 12, 679–686. 10.1007/s10008-007-0401-6

[B29] Steichen M. Dale P. (2011). Synthesis of trigonal selenium nanorods by electrodeposition from an ionic liquid at high temperature. Electrochem. Commun. 13, 865–868. 10.1016/j.elecom.2011.05.023

[B30] Tian Q. Deng D. Zhang Z. Li Y. Yang Y. Guo X. (2017). Facile synthesis of Ag2Se quantum dots and their application in Dye/Ag2Se co-sensitized solar cells. J. Mater. Sci. 52, 12131–12140. 10.1007/s10853-017-1366-1

[B31] Tsiulyanu D. Marian S. Miron V. Liess H. D. (2001). High sensitive tellurium based NO2 gas sensor. Sensors Actuators B 73, 35–39. 10.1016/S0925-4005(00)00659-6

[B32] Wei C. Myung N. Rajeshwar K. (1994). A combined voltammetry and electrochemical quartz crystal microgravimetry study of the reduction of aqueous Se(IV) at gold. J. Electroanal. Chem. 375, 109–115. 10.1016/0022-0728(94)03377-3

[B33] Xiong S. Xi B. Wang W. Wang C. Fei L. Zhou H. (2006). The fabrication and characterization of single-crystalline selenium nanoneedles. Cryst. Growth Des. 6, 1711–1716. 10.1021/cg060005t

[B34] Yan S. Wang H. Zhang Y. Li S. Xiao Z. (2009). Direct solution-phase synthesis of Se submicrotubes using Se powder as selenium source. Mater. Chem. Phys 114, 300–303. 10.1016/j.matchemphys.2008.09.013

[B35] Yuho M. Ho Jun S. Jong-Jin C. Byung-Dong H. Geon Dae M. (2018). Dimensional and compositional change of 1D chalcogen nanostructures leading to tunable localized surface plasmon resonances. Nanotechnology 29:345603. 29848801 10.1088/1361-6528/aac929

[B36] Zeng K. Chen S. Song Y. Li H. Li F. Liu P. (2013). Solvothermal synthesis of trigonal selenium with butterfly-like microstructure. Particuology 11, 614–617. 10.1016/j.partic.2012.06.007

[B37] Zhang Q. Li H. Ma Y. Zhai T. (2016). ZnSe nanostructures: synthesis, properties and applications. Prog. Mater. Sci. 83, 472–535. 10.1016/j.pmatsci.2016.07.005

[B38] Zhao L.-D. Lo S.-H. Zhang Y. Sun H. Tan G. Uher C.. (2014). Ultralow thermal conductivity and high thermoelectric figure of merit in SnSe crystals. Nature 508, 373–377. 10.1038/nature13184 24740068

[B39] Zhu Y.-J. Hu X.-L. (2004). Preparation of powders of selenium nanorods and nanowires by microwave-polyol method. Mater. Lett. 58, 1234–1236. 10.1016/j.matlet.2003.09.044

