# Supplementary material for: Template-Free Electrochemical Deposition of t-Se Nano- and Sub-micro Structures With Controlled Morphology and Dimensions
Source: Front Chem. 2020 Aug 31;8:785. doi: 10.3389/fchem.2020.00785 (PMC7493628; doi:10.3389/fchem.2020.00785)
Supplement: Supplementary file 1 [file Image_1.pdf]

Supplementary Material

1 Supplementary Data

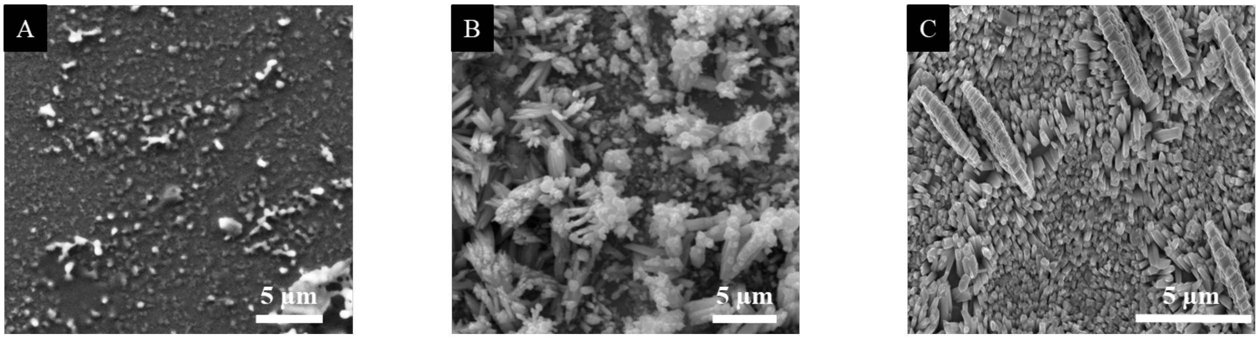

Fig. A1. SEM images of selenium electrodeposited for 150 minutes at (A) 25℃, (B) 60 ℃, and (C) 80℃. Applied potential and SeO_2 precursor concentration were fixed at -0.389 V and 100 mM, respectively.

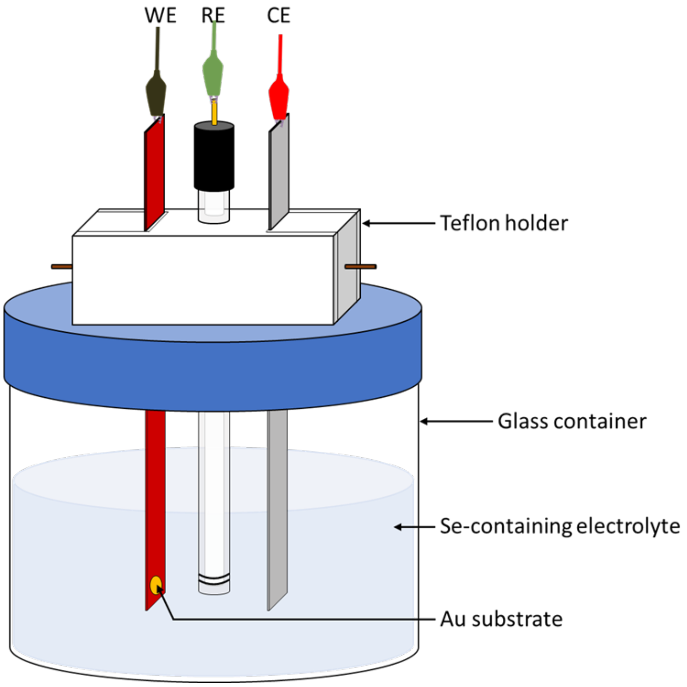

Fig. A2. Schematic of three-electrode electrochemical cell used for Se electrodeposition. Au substrate, which was used as the working electrode, had an area of 0.79 cm^2. Aluminum foil was used to cover the cell to prevent photoexcitation of selenium by light exposure. See the Experimental section for more details on the setup.

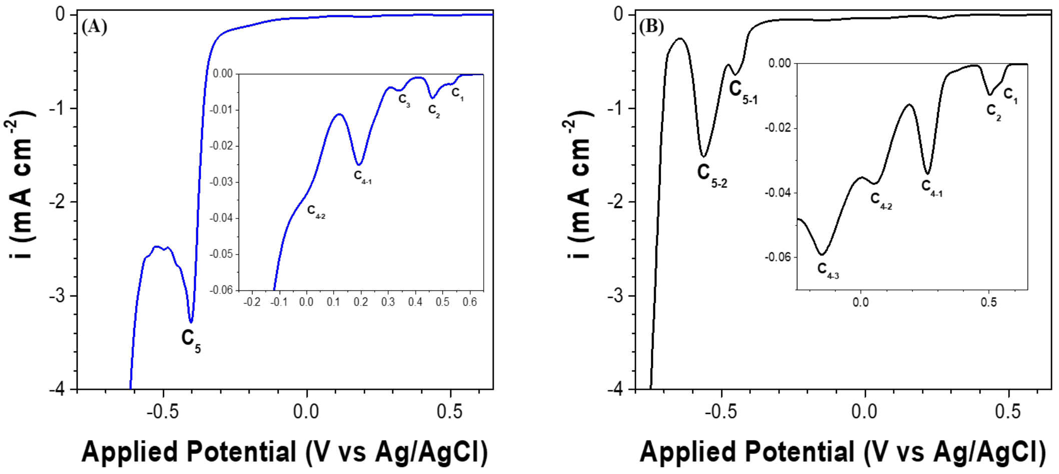

Figure A3. Linear sweep voltammograms (LSV) of Se electrodeposition from 0.65 to -0.8 V in electrolytes containing (A) 10 mM, and (B) 100 mM SeO_2 at pH of 1.5 and scan rate of 2 mV/s. Temperature was fixed at 80 ^oC with no illumination. Inset shows the expanded y-scale of applied potential from 0.65 to -0.25 V.

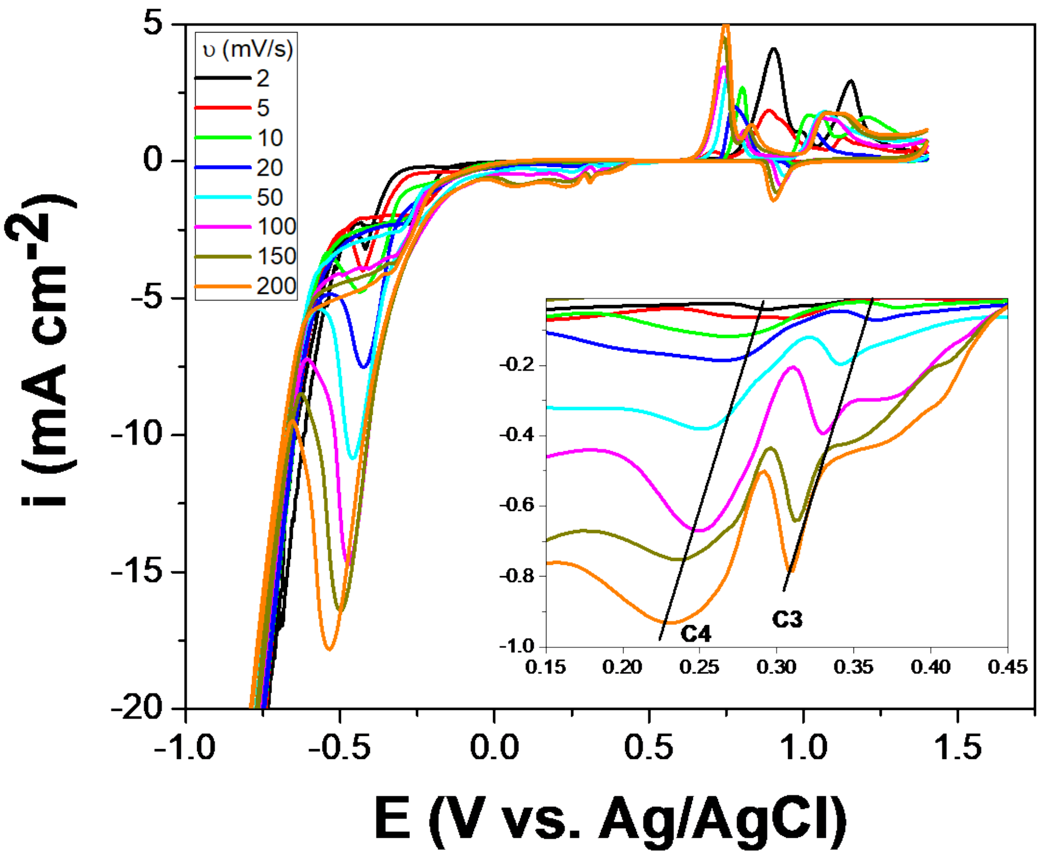

Fig. A4. Cyclic voltammograms (CV) of Se electrodeposition with different scan rates at pH of 1.5 and precursor concentration of 10 mM. Temperature was fixed at 80 °C. Inset shows expanded y-scale of applied potential from 0.45 to 0.15 V, with peak C3 and C4 identified for all scan rates.

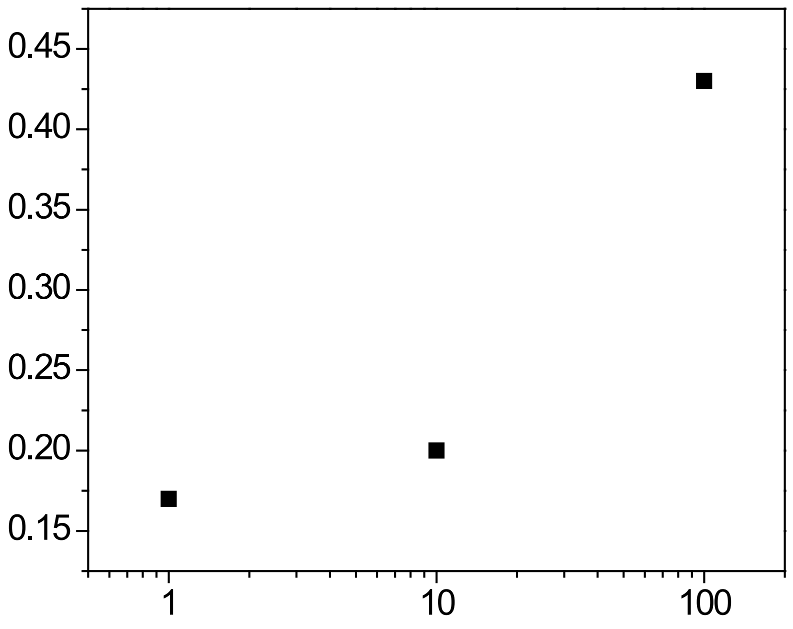

Fig. A5. Surface coverage of selenium corresponding to the underpotential deposition as a function of concentration. Surface coverage was calculated based on peaks C1-C3 obtained in LSV.

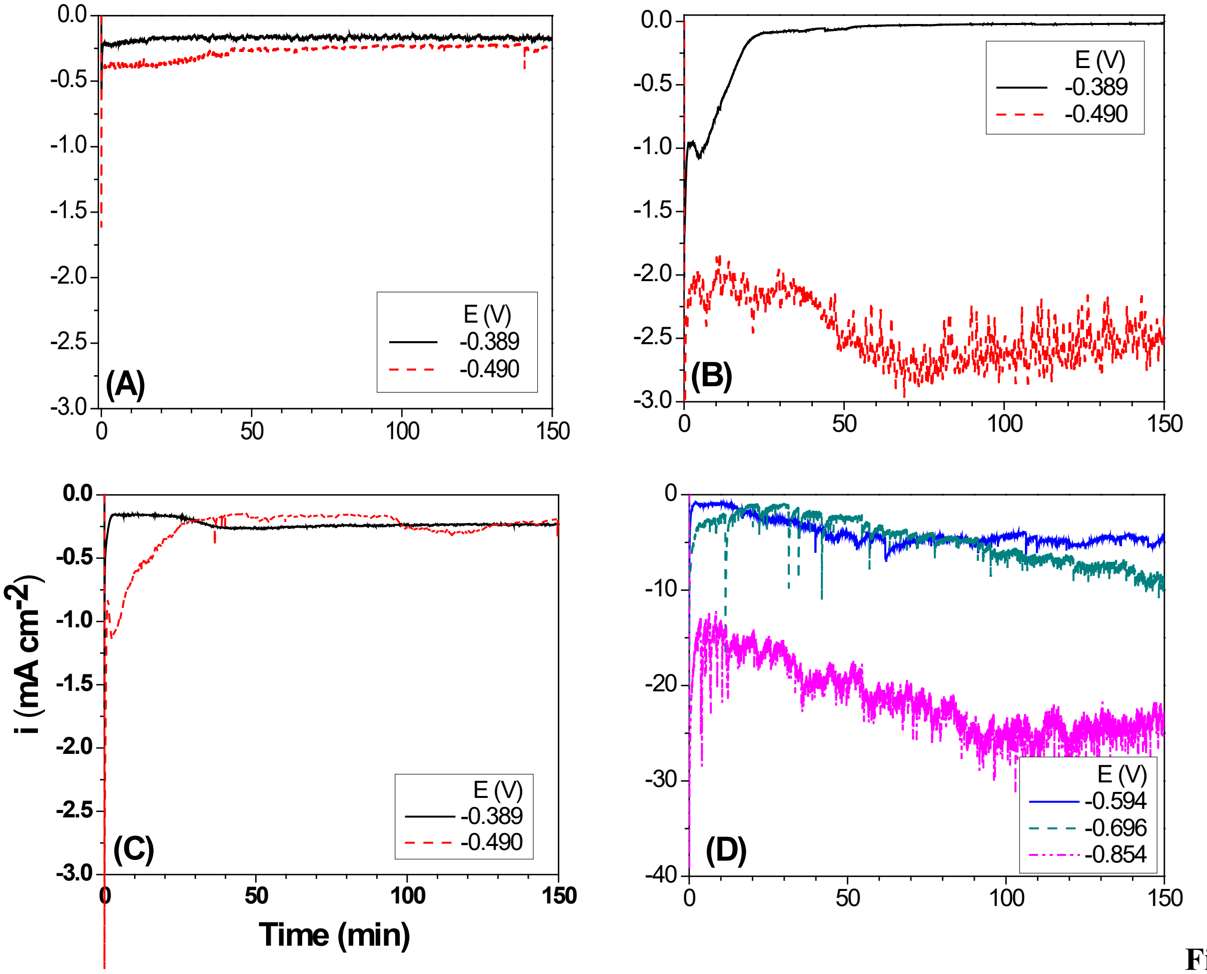

Fig. A6. Chronoamperogram of electrolyte containing (A) 1mM, (B) 10 mM, (C, D) 100 mM SeO_2 at different applied potentials after 150 minutes. Electrolyte temperature and pH were fixed at 80 °C and 1.5, respectively.

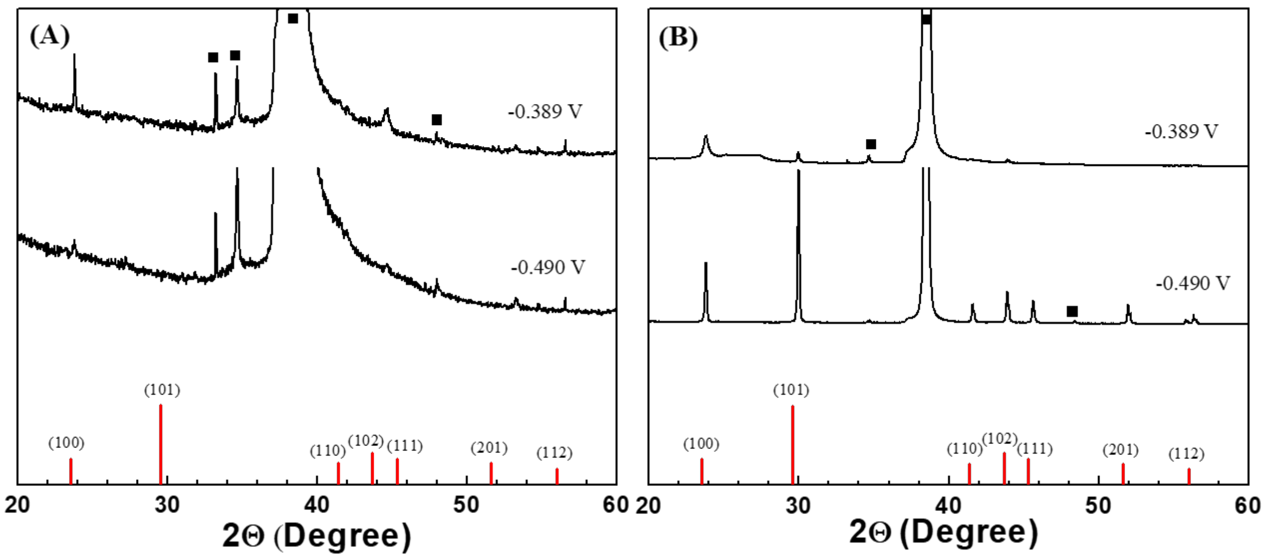

Fig. A7. XRD pattern of selenium electrodeposited from (A) 1 mM and (B) 10 mM SeO_2. The deposition time and temperature were fixed at 150 min and 80 ºC, respectively. The squares indicate peaks associated with the substrate.
